# Buildings Contemplated

**Published:** 1914-05-02

**Authors:** 


### Buildings Contemplated.

Burnley.—The Committee of the Victoria Hospital for Burnley and District are inviting architects to submit schemes for additions to the existing buildings. Those desirous of competing are asked to send in their names to the Honorary Secretary before May 4. Mr. H. Percy Adams, F.R.I.B.A., has been appointed assessor, and premiums of £75, £50, and £25 respectively will be awarded to those who are placed second, third, and fourth in the competition.

Chartham Downs.—The Kent County Asylums Committee invite tenders for the proposed additions to the Lunatic Asylum at Chartham Downs, near Canterbury. Bills of quantities may be obtained from the architect, Mr. W. J. Jennings, 4 St. Margaret's Street, Canterbury.

Dartford.—The Metropolitan Asylums Board invite tenders for the erection of certain buildings and the extension of others. Mr. W. T. Hatch, M.Inst.C.E., M.I.Mech.E., engineer-in-chief, is the architect. Forms of tender may be obtained at the office of the Board, Embankment, E.C.

Marylebone.—The Marylebone Board of Guardians invite tenders for alterations to several wards at the workhouse. Mr. A. Saxon Snell, F.R.I.B.A., 9 Bentinck Street, Manchester Square, W., is the architect. Specifications, drawings, and forms of tender may be obtained upon application to Mr. Henry T. Dudman, Clerk to the Board, Guardians' Offices, Northumberland Street, Marylebone Road, W.

